# Translation and Cultural Adaptation of the Turkish Lysholm Knee Scale: Ease of Use, Validity, and Reliability

**DOI:** 10.1007/s11999-013-3046-z

**Published:** 2013-05-11

**Authors:** Derya Celik, Dilber Coşkunsu, Önder Kılıçoğlu

**Affiliations:** 1 Division of Physiotherapy and Rehabilitation, Faculty of Health Sciences, Istanbul University, 34740 Bakırkoy, Istanbul, Turkey; 2 Sports Medicine Centre, Fulya Acıbadem Hospital, Istanbul, Turkey; 3 Department of Orthopaedics and Traumatology, Istanbul Faculty of Medicine, Istanbul University, Istanbul, Turkey

## Abstract

**Background:**

The Lysholm knee scale, first published in 1982, is an eight-item questionnaire designed to evaluate patients after knee ligament injury. However, as a tool developed in English, its use as a validated instrument has been limited to English-language populations.

**Questions/purposes:**

The objectives of this study were to test the ease of use, reliability, and validity of a Turkish-language, culturally adapted version of the Lysholm knee scale.

**Methods:**

The Lysholm knee scale was translated into Turkish according to Guillemin’s recommendations. Seventy patients (mean age, 36 years; range, 17–72 years) with different knee complaints were included, and the scale was completed twice by each participant at 3- to 14-day intervals to assess test-retest reliability based on the interrater correlation coefficient, whereas Cronbach’s alpha evaluated internal consistency. External validity was evaluated with correlations between the Lysholm knee scale, Kujala Anterior Knee Pain Scale, and SF-36. The distribution of floor and ceiling effects was determined.

**Results:**

Patients completed the Turkish-language Lysholm questionnaire in approximately 3 minutes. The test-retest reliability was 0.82, with a Cronbach’s alpha coefficient of 0.68. The Lysholm knee score was strongly correlated with the Kujala Anterior Knee Pain Scale (r = 0.78). The Turkish Lysholm knee scale showed high correlations with the SF-36 physical component score (r = 0.61) and a low association with the mental component domain (r = 0.14).

**Conclusions:**

The Turkish version of the Lysholm knee scale is quickly administered, valid, and reliable, and can be used for patients with various knee disorders.

**Level of Evidence:**

Level III, diagnostic study. See Guidelines for Authors for a complete description of levels of evidence.

## Introduction

Knee injuries are common in young individuals and usually result in pain or some degree of loss in athletic capacities [12]. These sequelae can be quantified or measured using knee scores or scales, such as the International Knee Documentation Committee (IKDC) Subjective Knee Form [13], Tegner Activity Scale [35], Marx Activity Rating Scale [24], Kujala Anterior Knee Pain Scale [18], and Knee Injury and Osteoarthritis Outcome Score (KOOS) [34]. These scores are based mainly on clinical findings, subjective complaints of the patients, or combinations of these factors. In general, assessments are categorized into two groups: general health and disease- or joint-specific indices. Before using such an outcome measurement in a community, they need to be translated and culturally adapted, given that the majority of these scores reflect the characteristics of the language and the social culture of the community in which they were established.

Most questionnaires in the orthopaedic literature were developed in English and therefore may reflect the Anglo-Saxon culture from which they derive. Many already are used as standards for the world scientific community, yet the appropriate use of these tools depends on their adaptation to different languages and cultures while maintaining cultural equivalence [28, 32]. To avoid the potentially confusing distribution of new questionnaires that are not comparable to those available in the literature, a rigorous adaptation process is needed, and mere translation is not sufficient. The availability of culturally equivalent outcome measures allows for multicenter studies to be reliably conducted across different countries. Questionnaires with valid Turkish versions include the KOOS [29], WOMAC [36], The Kujala Anterior Knee Pain Scale [19], and the Knee Outcome Survey-Activities for Daily Living Scale [9].

First published in 1982, the Lysholm knee scale was developed to determine the functional status of patients with anterior cruciate ligament injuries of the knee [21]. The questionnaire also has been validated for evaluation of patients with patellofemoral pain syndrome, patellar tendinitis, meniscal injuries, and various other traumatic and degenerative chondral lesions [2, 16, 22]. Compared with other Turkish-validated knee indices, the Lysholm knee scale shows distinct advantages. The WOMAC osteoarthritis index is a validated questionnaire used to assess symptoms and physical functional disability in patients with osteoarthritis of the knee and the hip [1]. In contrast, the KOOS [34] was developed as an extension of the WOMAC osteoarthritis index to evaluate short- and long-term symptoms and function in individuals with knee injuries and osteoarthritis. These two outcome scores also require considerable time to be completed. The KOOS and WOMAC require between 5 to 10 minutes to be completed [7]. The Knee Outcome Survey-Activities for Daily Living Scale [15] preferentially focuses on daily living activities rather than the patients’ symptoms. However, the Lysholm knee scale is more concise and therefore requires little time for the patient to complete and for the health practitioner to evaluate. Furthermore, it is not disease-specific and therefore can be used to evaluate various knee disorders [2, 16, 22].

Although the psychometric properties of the Lysholm knee scale in relation to various knee disorders have been reported [2–5, 13, 16, 22, 30, 33], to our knowledge, only validated Portuguese and German translations of the Lysholm knee scale study have been published [31, 38]. Therefore, the objectives of our study were to evaluate the ease of use, reliability, and validity of a Turkish-language, culturally adapted version of the Lysholm knee scale.

## Patients and Methods

We translated the Lysholm knee scale into Turkish and culturally adapted the items in accordance with the stages recommended by Guillemin [10]. Two Turkish individuals [EKM, AO] with a strong command of English were responsible for the literary and conceptual translation of the Lysholm knee scale, including a physical therapist [EKM] acting as the informed translator and a teacher [AO] acting as the uninformed translator. Both translators’ native language is Turkish and both are fluent in English. The translations were completed independently, and both translations then were compared and reviewed by a bilingual individual [GK] who highlighted any conceptual errors or inconsistencies identified in the translations. Once the primary Turkish translation was established, two native English speakers [DU, DA] with a good command of Turkish independently back-translated the finalized Turkish translation into English. Both of these translators were unaware of the purpose of the study and had no access to the original scale. The subsequent versions of the questionnaire were compared with the initial translation. A committee consisting of four translators [DC, OK, GK, CO] compared the English retranslation with the initial Turkish translation before approving the Turkish version of the Lysholm knee scale. Once approved, the assessment form was administered to the patients. The pilot study was conducted with 20 patients.

The Lysholm knee scale is an eight-item questionnaire designed to evaluate patients after knee ligament injury. It is scored on a 100-point scale from 0 to 100 (worst to best symptoms, respectively), with 25 points attributed to pain, 15 to locking, 10 to swelling, 25 to instability, 10 to stair climbing, and 5 points each to limping, use of a support, and squatting [21].

The Kujala Anterior Knee Pain Scale developed by Kujala et al. [18] is comprised of 13 questions that inquire about the following factors: pain (with walking up and down stairs, squatting, running, jumping, or prolonged sitting with the knee in flexion); whether there is limping, swelling, or subluxation of the patella; the amount of atrophy in the quadriceps muscle, flexion deficiency, and pain; and whether a walking assist is needed. The total score ranges from 0 to 100, with the highest value indicating the best score [18].

The SF-36 consisting of eight scaled scores was used to establish a health profile, and each scale was directly transformed into a score from 0 to 100 to identify the patient’s physical and mental state [36]. These eight sections included physical functioning, physical role functioning, bodily pain, general health perceptions, vitality, social role functioning, emotional role functioning, and mental health [37].

Eighty patients with knee complaints were recruited from the Istanbul University Faculty of Medicine’s Department of Orthopedics and Fulya Acıbadem Hospital between March 2011 and January 2012. As the oldest and largest hospital in Istanbul, Istanbul Medical School attracts patients from all cultures and income levels. However, high-level athletes and patients with very high incomes prefer private hospitals. Therefore, to include a diverse population of patients, some patients were recruited from one of the private hospitals. Some data were excluded for the following reasons: five patients did not return for the retest assessment, two declined to complete the SF-36, and three had received medical treatment before the retest assessment, thereby rendering the retest potentially unreliable. In total, 70 patients (37 males, 33 females; mean age, 36.10 ± 13.5 years; range, 17–72 years) with knee complaints were included in the study (Table 1).

**Table 1:** Patient demographics

Demographic	Number (%)
Sex	
Female	33 (47.1)
Male	37 (52.9)
Education	
Primary school	15 (21.4)
High school	16 (22.9)
College student	6 (8.5)
University degree	27 (38.5)
Master’s degree	4 (5.7)
Doctorate	2 (2.9)
Dominant/nondominant side	
Involved right knee	34 (48.5)
Involved left knee	26 (37.1)
Involved both knees	10 (14.2)
Diagnosis	
ACL injury	12 (17.1)
ACL repair	14 (20)
ACL and lateral meniscus lesion	5 (7.1)
ACL and meniscus repair	5 (7.1)
Meniscus injury	4 (5.7)
Meniscectomy	4 (5.7)
Patellofemoral pain syndrome	20 (28.5)
Patellar dislocation	1 (1.4)
Multiple ligament injury	1 (1.4)
Osteoarthritis	4 (5.7)

ACL = anterior cruciate ligament.

The inclusion criteria were: (1) 16 years or older; (2) presence of a knee problem; and (3) no treatment between the test-retest assessments. The exclusion criteria were: (1) inability to complete the forms as a result of cognitive impairment; (2) illiteracy or lack of understanding of Turkish; (3) had a condition that could not be stabilized after a second assessment for other ailments such as cancer, serious infection, or inflammatory disease; and (4) the presence of neurologic or musculoskeletal disorders other than the knee condition. The duration of patients’ knee symptoms in the group surveyed was 5.3 ± 4.2 months.

Patients were examined clinically by two experienced knee surgeons (OK, OT). When necessary, radiography and MRI were performed. Before inclusion in the study group, participants provided written informed consent, which had been approved by the ethical committee at Istanbul University (IRB study protocol: 2010/875-265).

Patients were asked to complete the Turkish version of the Lysholm knee scale (Appendix), the previously validated Turkish version of the Kujala Anterior Knee Pain Scale [19], and the Turkish SF-36 [17].

Physical therapists administered the listed questionnaires to patients in waiting rooms before their appointments with an orthopaedic surgeon. After each patient completed the questionnaire, physical therapists checked for missing responses. Patients who skipped a question on the questionnaire were asked for the reason. The difficulty in understanding the question or the incompatibility with their problem was noted and the time required to complete the questionnaires was recorded.

Ease of use was measured by the time it took to complete the questionnaire. Additionally, we documented problems with comprehension of particular translated terms as they arose.

The test-retest reliability, which is a measure of stability or reproducibility, represents a scale’s capability of providing consistent results when administered on separate occasions [8, 23]. The reliability of scale scores has been estimated using the internal consistency method and test-retest method across repeated administrations. To determine the test-retest reliability, 70 patients were asked to complete the Lysholm scale 3 to 14 days after the first assessment. To minimize the risk of short-term clinical change, no treatments were provided during this period.

Validity is represented by the extent to which a score retains its intended meaning and interpretation [20]. In our study, validity was assessed according to the following three factors: construct, convergent and divergent, and content validity. The construct validity of the Turkish Lysholm knee scale was analyzed based on its correlation with the Kujala Anterior Knee Pain Scale and the physical component score of the SF-36. Correlations with the physical functioning, physical role functioning, and physical component score domains were used to assess the convergent validity. Divergent validity was evaluated using the SF-36 mental health, emotional role functioning, and mental component score domains. It was hypothesized that the physical domains of the SF-36 would correlate more closely with disease- or joint-specific questionnaires compared with the mental domains. Content validity was assessed using the distribution and the occurrence of ceiling and floor effects. A ceiling effect occurs when the maximum possible score of 100 is achieved, whereas a floor effect is observed when the minimum possible score of 0 is reached. We considered scores between 90% and 100% to be maximum scores and scores between 0% and 10% were minimum scores. If more than 15% of the patients scored maximum or minimum scores, we considered these to be floor and ceiling effects, respectively.

The intraclass correlation coefficient (ICC) was used to measure the test-retest reliability of the Lysholm knee scale assessment form. Correlation values of 0.4 or greater were considered satisfactory (specifically, r ≥ 0.81–1.0 was excellent, 0.61–0.80 was very good, 0.41–0.60 was good, 0.21–0.40 was fair, and 0.00–0.20 was poor) [14, 20]. Cronbach’s alpha coefficient was used to determine internal consistency. Construct and convergent and divergent validities were evaluated with Pearson’s correlation coefficients, and a 95% CI was used for all correlation coefficients. A Student’s paired t-test was used to detect statistically significant differences between the first and repeat tests.

All statistical analyses were performed with the Statistical Package for the Social Sciences (SPSS) 17.5 (SPSS Inc, Chicago, IL, USA). The agreed level of significance was p less than 0.05. Floor and ceiling effects and the number of items answered were identical during the test and retest examinations.

## Results

The Lysholm knee scale required approximately 3 minutes to complete. The pilot study was conducted with 20 patients (seven females, 13 males; mean age, 35.00 ± 12.5 years; range, 17–60 years). Some patients had difficulty answering the pain subscale items because individuals in Turkey usually use minutes rather than kilometers to estimate walking distances. Therefore, we parenthetically indicated that 30 minutes equaled 2 km. Another difficulty was encountered when checking the items left blank by the patients. Two patients did not answer the question regarding instability. “Boşalma” is the most appropriate Turkish word for translating the term “instability,’’ yet some of the patients still experienced difficulty understanding this term. However, the linguistic committee could not find a more appropriate word to replace it.

The Turkish-language Lysholm questionnaire showed adequate reliability.

Cronbach’s alpha value was calculated as 0.68. The Lysholm knee scoring domains and the total score exhibited good to excellent ICC values. The paired t-test did not show any statistically significant difference between the test-retest means (Table 2) based on the mean test interval of 5.4 ± 2.2 days.

**Table 2:** Test-retest reliability of the Lysholm knee scale

Lysholm knee scale	Mean ± SD		Reliability	
	Test 1	Test 2	p values	Intraclass correlation coefficient
Pain	11.78 ± 11.1	13.63 ± 10.8	< 0.001	0.72
Instability	17.90 ± 7.1	17.88 ± 6.8	< 0.001	0.71
Locking	11.68 ± 3.7	11.37 ± 4.0	< 0.001	0.84
Stair climbing	6.85 ± 3.0	6.71 ± 4.2	< 0.001	0.49
Limp	3.73 ± 1.9	3.76 ± 1.4	< 0.001	0.81
Support	5.05 ± 0.9	4.88 ± 0.5	0.030	0.50
Swelling	7.30 ± 4.4	7.50 ± 4.4	< 0.001	0.96
Squatting	3.20 ± 2.3	2.83 ± 1.8	< 0.001	0.64
Overall Lysholm knee scale	69.33 ± 20.6	67.71 ± 19.0	< 0.001	0.82

The Turkish-language questionnaire showed good to excellent validity. The correlation coefficient between the Lysholm knee scale and the Kujala Anterior Knee Pain Scale score was 0.78, which is considered extremely strong (p < 0.001). The highest correlation was found between the Lysholm knee scale and SF-36 physical function, the SF-36 bodily pain, and the Lysholm knee scale and physical component score (r = 0.61, r = 0.55, r = 0.56, respectively; p < 0.001). Conversely, the lowest correlations were identified between the Lysholm knee scale and the SF-36 social function, and between the Lysholm knee scale and the SF-36 mental component score (r = 0.23 and r = 0.14, respectively; p < 0.001) (Table 3). Ten of 70 patients (14%) scored between 90 and 100 so there was a slight ceiling effect. There were no floor effects because none of the patients scored between 0 and 10.

**Table 3:** Comparison of SF-36 subscale results and overall Lysholm knee scale

SF-36 subscale	Overall Lysholm knee scale score			
	Current study	Marx et al. [22]	Paxton et al. [30]	Kocher et al. [16]
SF-36 (PF)	0.61*	0.66	0.57	0.53
SF-36 (RP)	0.43*	0.49	0.38	0.47
SF-36 (BP)	0.55*	0.57	0.50	0.55
SF-36 (GH)	0.41*	0.31		
SF-36 (VT)	0.38	0.28		
p value	0.01			
SF-36 (SF)	0.24	0.50		
p value	0.04			
SF-36 (RE)	0.34	0.18		
p value	0.04			
SF-36 (MH)	0.24	0.29		
p value	0.04			
SF-36 (PCS)	0.57*	0.68		
SF-36 (MCS)	0.14	0.05		
p value	0.24			

* p < 0.000; PF = physical functioning; RP = physical role functioning; BP = bodily pain; GH = general health perceptions; VT = vitality; SF = social function; RE = emotional role functioning; MH = mental health; PCS = physical component scale; MCS = mental component scale.

## Discussion

The primary outcome of this study is that the Turkish translation of the Lysholm knee scale was shown to be easy to use, reliable, and valid. Our findings show the acceptable psychometric performance of this scale for patients with various knee disorders in the Turkish population.

The primary limitations of our study included the untested statistical power and small sample size. However, previous validation studies have used similar numbers of individuals, and the sample size was large enough to reach statistical significance. Nevertheless, the Turkish Lysholm knee scale should be applied to larger populations to evaluate its reliability, validity, responsiveness, and minimal clinically important differences in patients with various diagnoses. In addition, there is no ideal or universally accepted interval for requerying patients regarding their health status. Short test-retest intervals carry the risk of patients “becoming familiar with the questions” and simply answering based on memory of the first assessment. Although longer intervals can decrease this possibility, other factors need to be considered to prevent bias in such studies. For instance, only patients with chronic, mostly degenerative disorders can be included in studies with long rescanning intervals because failing to treat an acute complaint for an extended period is unethical. Furthermore, spontaneous improvement of acute complaints may occur. Even in patients with chronic disorders, spontaneous changes in complaints can be observed. In general, the length of time between repeat administrations of a clinical outcome measure should be relatively short (3–7 days) when the measured condition is expected to change rapidly [20, 26]. In the literature, the reported intervals for retesting the Lysholm knee scale usually are longer than advised periods (Table 4). We selected an interval of 3 to 14 days, similar to previous studies [2, 11, 22, 27, 35], and thus the clinical limitations associated with this choice are acknowledged.

**Table 4:** Test-retest reliability, internal consistency, and validity of the Lysholm knee scale

Lysholm knee scale	Test-retest reliability (ICC)	Interval	Cronbach’s alpha	Correlations with Lysholm knee scale and other knee scores
Tegner and Lysholm [35]	0.90	2 weeks		
Bengston et al. [2]	0.75	1–3 days		
	0.69	1–14 days		
	0.68	3–14 days		
Irrgang et al. [13]			0.60– 0.73	
Risberg et al. [33]				Cincinnati 0.78–0.86
Marx et al. [22]	0.95	2–14 days		Cincinnati 0.70 AAOS 0.70 ADL 0.85
Paxton et al. [30]	0.88	21 days	0.71	Tegner 0.24 Fulkerson 0.93 Kujala 0.86
Kocher et al. [16]			0.65	
				WOMAC 0.65 Tegner 0.34
Overall score	0.91			
Pain	0.61			
Stair climbing	0.67			
Briggs et al. [3]	0.92	4 weeks	0.72	
Briggs et al. [4]	0.94	4 weeks	0.72	IKDC 0.78 Tegner 0.07 Kujala 0.78
Briggs et al. [5]				
Current study	0.82	3–14 days	0.68	

ICC = intraclass correlation coefficient; AAOS = American Academy of Orthopaedic Surgeons; ADL = activities of daily living; IKDC = International Knee Documentation Committee.

Although patients found the questionnaire easy and fast to complete, some issues arose with the translation and cultural adaptation. During the translation procedures, the translators could not agree on the ideal Turkish word for “instability.” Whereas most of the various meanings of “instability,” such as imbalance, lability, and variability, have a counterpart in the Turkish language, knee instability does not have a perfect equivalent. Instead, patients describe their knee instability with phrases such as “giving way”, “weakness”, or “insecurity”. The Turkish term “boşalma”, which means “giving way”, was found to be the most appropriate choice and was used for “instability” and “giving way”. For cultural adaptation purposes, the distance unit had to be changed to metric units, similar to previous translations of the Lower Extremity Functional Scale (LEFS) into the Brazilian Portuguese [25] and Italian languages [6]. Despite miles being adapted to kilometers, some patients still were unable to answer this question because they were unaccustomed to describing walking distance. Instead, they preferred to describe walking duration. This difficulty was observed even during the pilot tests. We initially tried to replace the distance unit with “bus station”, however, the patients felt more comfortable explaining distance as minutes spent walking. Therefore, we included distance and duration in the questionnaire.

The test-retest indicated adequate to excellent reliability for the subscales and the Turkish Lysholm knee scale as a whole (Table 2). In the literature, the test-retest reliability of the overall Lysholm knee scale typically has been excellent, with the exception of Bengston et al. [2] (Table 4). In considering subscales of the Lysholm knee scale, Kocher et al. found a reliability of 0.61 for pain and 0.67 for stair climbing subscales [16] (Table 4). In our study, the pain subscale was 0.72, but the stair climbing subscale showed less reliability (0.49). The test-retest reliability for the support domain also was less acceptable. Thus, these two domains (stair climbing and support) may lack the reproducibility necessary for scientific precision and require further refinement to improve their reliability. Cronbach’s alpha coefficient for the Lysholm knee scale was 0.68 in our study, which is questionable. However, its reliability was similar to that of other studies (Table 4) [4, 5, 16, 22, 30].

In some studies, the original Lysholm knee scale was compared with other outcome tools using multiple scores and scales [4, 5, 16, 22, 30, 33]. High correlation coefficients have been reported with the Fulkerson, Cincinnati Knee Rating System, and Kujala Anterior Knee Pain Scale, with the lowest correlation found with the Tegner activity scale (Table 4) [16, 30, 33]. In our study, the convergent validity was assessed by comparing the Lysholm knee scale with the Kujala Anterior Knee Pain Scale and the SF-36 questionnaire. The Kujala Anterior Knee Pain Scale was preferred for convergent validity because the subscales, such as pain, swelling, squatting, and stair climbing, of the Kujala Anterior Knee Pain Scale and Lysholm knee scale are similar. The correlation coefficient between the Lysholm knee scale and the Kujala Anterior Knee Pain Scale score was 0.78, which is considered good. The correlation between the Lysholm knee scale and SF-36 was variable, from fair to excellent. This range of results is not surprising and we believe are the result of contextual differences between condition-specific questionnaires, such as the IKDC, Western Ontario Meniscal Evaluation Tool (WOMET), and Cincinnati knee rating system. The strength of correlations between the SF-36 and scores of specific instruments have been limited. This confirms that the SF-36 measures additional aspects of physical health and therefore provides a more comprehensive, but less specific, range of information about a patient’s overall health than obtained with condition-specific questionnaires. Researchers have investigated the correlations between the Lysholm knee scale and subscales of the SF-36 across different settings; their results concluded poor to high correlations (Table 3) [4, 5, 16, 22, 30]. In our study, the correlation between the Lysholm knee scale and SF-36 physical function values was higher those reported by Paxton et al. [30] and Kocher et al. [16], but lower than that reported by Marx et al. [22]. The correlation between the SF-36 physical role function and bodily pain domains was similar to our results. Compared with the correlations of Marx et al. [22], the correlations we found among the SF-36 general health perceptions, vitality, and emotional role function subdomains were superior with the Lysholm knee scale. The correlation between the SF-36 physical component score and the Lysholm knee scale was higher in the study of Marx et al. [22]. The Turkish Lysholm knee scale contains an adequate number of questions to reveal the functional status and pain of the patients. It is short and easy to administer and interpret with a minimal amount of time required for clinicians, patients, or researchers. The Turkish translation and culturally adapted version are reliable and valid and can be used to assess the functional limitations of Turkish patients with knee disorders. Whereas the presented translation has been validated with this preliminary study, the Turkish form still should be validated in larger and more diverse populations.

## Appendix

**Table:** LYSHOLM DİZ SKALASI

Aksama (5 puan)	
Yok	5
Hafif veya belirli aralıklarla	3
Ciddi veya her zaman	0
Destek (5 puan)	
Yok	5
Baston veya koltuk değneği	2
Üzerine basmak imkansız	0
Kilitlenme (15 puan)	
Kilitlenme veya takılma hissi yok	15
Takılma hissi var fakat kilitlenme yok	10
Kilitlenme	
Bazen	6
Sık	2
Muayenede eklem kilitli	0
Boşalma (Dizin öne doğru kayması) (25 puan)	
Boşalma hissi hiç yok	25
Zorlayıcı veya sportif aktivitelerde bazen	20
Zorlayıcı veya sportif aktivitelerde sık	15
Günlük aktivitelerde bazen	10
Günlük aktivitelerde sık	5
Her adımda	0
Merdiven çıkma (10 puan)	
Problem yok	10
Hafif problem var	6
Tek bacak atarak	2
İmkansız	0
Çömelme (5 puan)	
Problem yok	5
Hafif problem var	4
90’den sonra problem var	2
İmkansız	0
Şişlik (10 puan)	
Yok	10
Zorlayıcı aktiviteler ile	6
Basit zorlanmalar ile	2
Devamlı	0
Ağrı (25 puan)	
Yok	25
Sürekli değil, zorlayıcı aktiviteler sırasında hafif	20
Zorlayıcı aktiviteler sırasında belirgin	0
2 km’den (30 dakika) fazla yüründüğünde belirgin	10
2 km’den (30 dakika) az yüründüğünde belirgin	5
Devamlı	0
Toplam puan	
